# Bone Tissue is an Integral Part of the Fascial System

**DOI:** 10.7759/cureus.3824

**Published:** 2019-01-03

**Authors:** Bruno Bordoni, Maria Marcella Lagana

**Affiliations:** 1 Cardiology, Foundation Don Carlo Gnocchi, Milan, ITA; 2 Radiology, Foundation Don Carlo Gnocchi, Milan, ITA

**Keywords:** fascia, myofascial, fascial system, bone

## Abstract

Bone tissue is not considered an integral part of the fascial system as per the current definition of fascia. Bodily fasciae derive from the mesoderm, while the fasciae associated with the cranial-cervical area derive from the ectoderm. Bone tissue or specialized connective tissue follows the same development process, but with a greater admixture between the two embryological sheets. Bone tissue is the largest organ capable of producing autocrine and paracrine substances, influencing its own metabolism and that of other organs. This article reviews the functions of bone, the anatomy that determines its shape, and its relationships within an organism. The objective of the article is to provide a scientific rationale for incorporating bone tissue within the definition of fascia, using the most up-to-date scientific knowledge.

## Introduction and background

No one definition of the fascial system has yet been accepted by all researchers. One of the most commonly used definitions derives from the Fascia Nomenclature Committee (2014), created by the Fascia Research Society and founded in 2007: “The fascial system includes adipose tissue, adventitia, neurovascular sheaths, aponeuroses, deep and superficial fasciae, dermis, epineurium, joint capsules, ligaments, membranes, meninges, myofascial expansions, periostea, retinacula, septa, tendons (including endotendon/peritendon/epitendon/paratendon), visceral fasciae, and all the intramuscular and intermuscular connective tissues, including endomysium/perimysium/epimysium [1].” The fascial system is an anatomical continuum that connects every part of the body. A recent study showed, for example, that the thoracolumbar fascia is in contact with the fascia of the abdominal muscles [2]. Microscopically speaking, we know that there are no discontinuations in the fascia because there is an absolute anatomical and functional continuity [3-4]. In a previous paper, we attempted to establish a new definition of the fascial system, viewing tissue from a functional and embryological point of view, including the epidermis and the bone tissue, which had been excluded from previous classifications: “The fascia is any tissue that contains features capable of responding to mechanical stimuli. The fascial continuum is the result of the evolution of the perfect synergy among different tissues, capable of supporting, dividing, penetrating and connecting all the districts of the body, from the epidermis to the bone, involving all the functions and organic structures. The continuum constantly transmits and receives mechano-metabolic information that can influence the shape and function of the entire body. These afferent/efferent impulses come from the fascia and the tissues that are not considered as part of the fascia in a biunivocal mode [5].” In addition to the solid state of the fascia, we recently tried to introduce the concept of a liquid fascia, that is, a specialized connective tissue that constitutes an integral part of the fascial system: blood and lymph. To do this, we developed a new theoretical model incorporating liquids into the biotensegretive vision: Rapid Adaptability of Internal Network (RAIN) [6]. Currently, only the periosteum, a connective tissue sheath (dense connective tissue) that covers the richly vascularized bone, is considered by scholars as an integral part of the fascial system. It is divided into two layers. The first, outer layer is rich in vascular and nerve vessels, fibroblasts and elastin, and collagen; this layer determines the mechanical stability of the periosteum [7]. The second, deepest layer consists of osteoblasts, smaller fibroblasts and a homogeneous diameter (isodiametric), with adult mesenchymal skeletal progenitor cells; this layer is fundamental for regenerative processes [7]. This article reviews bone tissue in terms of its local and systemic functions, with the aim of developing a potential new definition of fascia, as bone is a connective tissue.

## Review

Embryological derivation of bone tissue

Bodily fasciae derive from the mesoderm, while the fasciae associated with the cranial-cervical area derive from the ectoderm [5]. Bone tissue or specialized connective tissue follows the same development process, but with a greater admixture between the two embryological sheets. The bones of the skull and the first cervical vertebrae originate from the mesoderm and the ectoderm. To give examples of the bones of the skull, the sphenoid bone (the orbitosphenoid and basal-post-sphenoid portion) originates from the cephalic mesoderm and neural crest cells (the alisphenoid and basal-pre-sphenoid portion) [8]. The occipital bone is formed from the paraxial mesoderm (basiocciput, jugular tubercles, foramen magnum, anterior tubercle of the clivus, occipital condyles) and from the neural crests of the notochord (the remaining parts of the occipital bone) [9-10]. The facial bones or splanchnocranium derive mainly from the cells of the ectoderm, except for some parts of the mandible (mesoderm); the bones of the cranial vault originate from both the ectoderm (frontal bone) and the endoderm (parietal bones) [11-12]. The first vertebra in particular develops from the notochord, while the remaining part of the vertebral column derives from the paraxial mesoderm as well as the ribs and the scapula [10,13]. The sternal bone derives from the lateral plate of the mesoderm; the cells migrate from a different area of the mesoderm, laterally towards the center of the mesoderm [14]. The bones that will constitute the limbs derive from the lateral plate of the mesoderm [15].

Bone tissue is an organ

Bone is traditionally regarded as a target for different hormonal substances (1.25 dihydroxy vitamin D, calcitonin, sex hormones and growth hormones, thyroid hormones), growth factors [transforming growth factor beta (TGF-B), insulin-like growth factor (IGF-1), fibroblast growth factor (FGF), bone morphogenic proteins (BMPs), and platelet-derived growth factor (PDGF)], as well as inflammatory substances [interleukins (IL-1β, IL-6) and tumor necrosis factor α (TNF-α)] [16-18]. Bone tissue is the largest organ capable of producing autocrine and paracrine substances, influencing its own metabolism and that of other organs [16]. The osteocyte is the most abundant bone cell capable of secreting sclerostin; the latter influences bone metabolism (autocrine action) and systemic metabolism (paracrine action) [16]. An increase in blood sclerostin is found in particular when the bone has a decreased stimulation to the load. Autocrine action stimulates a minor remodeling of the bone (osteoporosis), while paracrine action influences insulin action [16]. Another molecule produced by osteocytes is a phosphatonin, more precisely, fibroblast growth factor 23 (FGF23). The transmembrane receptor of FGF23 (a protein known as Klotho) is found in the osteocytes and other tissues, such as the thyroid gland and the kidneys [16]. A reduced level of FGF23 and its Klotho receptor is correlated with premature aging and systemic endothelial dysfunction, while an optimal level of FGF23 positively influences renal function, protecting the kidney from phosphate retention and excessive production of parathormone [16]. An excess of FGF23 is detrimental to the health of the central nervous system. FGF23 is also associated with alcohol abuse, and increased FGF23 beyond normal physiological threshold values causes an alteration in hippocampal morphology, and a cognitive decline [16]. Osteocalcin, a peptidic hormone synthesized by osteoblasts, is essential for optimal adaptation of muscle fibers after exercise, probably owing to an increase in insulin sensitivity in myofibers [16]. It is able to stimulate, through a membrane receptor (G protein-coupled receptor family C group 6 member A, GPRC6A), the production of insulin from the pancreatic beta cells, and influence the lipid metabolism of the liver [16]. A recent study using an animal model demonstrated the ability of osteocalcin/GPRC6A to stimulate the synthesis of luteinizing hormone (LH), as well as the production and release of testosterone from Leydig cells. In this way, bone can control male hormones, utilizing a path independent of the hypothalamus-pituitary axis [16]. Bone tissue is fundamental for the general health of the individual, influencing different organs and systems, through the hormonal paracrine production of bone cells [17-18].

Bone tissue cells

Adult bone contains three major cell types: the osteocyte, which accounts for about 90%–95% of all bone cells; the osteoblast, which derives from mesenchymal stem cells; and the osteoclast, which derives from hematopoietic progenitor cells [19]. Osteocytes develop from the osteoblasts and are found in the bone matrix and on the bone surface. They are considered essential for maintaining bone turnover, through the production of the sclerostin protein and its receptor [nuclear factor (NF)-kB ligand and receptor activator of NF-kΒ ligand (RANKL)] [19]. Osteocytes control the activity of osteoblasts (which create and repair bone tissue) and osteoclasts (which disassemble bone tissue), allowing the bone to adapt and responding to any mechano-metabolic stimuli [19]. Osteocytes are the main sensors of mechanical stimuli. The osteocytes form a network within the entire bone tissue (lacunar-canalicular), so that any stimulus can be transported and sensed by the entire bone area; mechanical energy is converted into electrical energy or a biochemical signal [20]. This transductive mechanism of the osteocyte is favored by the Wnt (canonical pathway) biochemical pathway, involving proteins that help to transport the signal inward [21]. Each osteocyte senses what is happening to the entire bone, owing to the presence of junction or gap-junction proteins, in particular, connexin-43; together, they constitute the osteocyte or lacunar-canalicular network [20]. How are mechanical signals, with respect to the whole bone, managed by the cells? The osteocytes are dispersed throughout the bone matrix, which consists of type I collagen (and other noncollagenous proteins such as osteopontin, bone sialoproteins, proteoglycans), minerals (carbonated apatite crystals), and water [22]. The osteocytes act as mechanical sensors, but water also plays a fundamental role in the transduction of the mechanical signal [22]. At the ultrastructural level (nanomillimeters) of bone are collagen fibrils and hydroxyapatite crystals, whose bonds constitute a large part of the matrix; this bond makes it possible to create a state of pre-tension [22-23]. When the mechanical message to the bone is transmitted through the osteocyte via Wnt, the bone matrix becomes deformed, generating electrical charges or movements of water (fluid flow shear stress). The water moves through the entire bone, deforming the structures along its passage, due to slippage between the collagen fibers and the hydroxyapatite crystals (bone elasticity), interacting simultaneously with all the osteocytes [22-23]. Hydration is an important component for the proper maintenance of bone tissue; water represents about 15%–25% of the total volume of bone, thereby establishing a variable pressure gradient [24-25].

Vascular system and bone innervation

Inside the matrix in the cortical area (the innermost layer of bone) are pores known as Haversian canals, located inside the lacunar-canalicular network (also called “active osteonal bone”) [26]. In these Haversian channels, surrounded by a thin bony lamella (known as a cement line), we find the blood and nerve vessels [25]. Haversian channels have a longitudinal pattern but lie at an angle of about 15–30 degrees from the median line of the bone [26-27]. Volkmann canals, positioned transversely, connect the Haversian canals, creating a shared blood network [27]. The entire bone system is richly vascularized, from the bone marrow to the periosteum [27]. It is the heart and the movements of the muscles that enable the entry and exit of blood to and from the bones [27]. The function of the lymphatic system inside the bone is not clear; it is probable that waste metabolites are transported by the outgoing venous system [27]. Bone is innervated by parasympathetic fibers, which communicate with acetylcholine (Ach) bone receptors, contributing to bone growth. Vagal innervation to bone is induced via stimulation of the central nervous system by interleukin-1; in this modality, the vagus stimulates apoptosis of the osteoclasts [28]. Precise data on the topographic presence of the vagus nerve in bone are lacking. Bone is also affected by innervation of the sympathetic system, with a more complex penetration of the tissue, involving the cortical area and the bone marrow that occurs with the parasympathetic system [29]. The sympathetic activation of the bone suppresses bone growth, stimulating the activity and production of osteoclasts, which are released from nerve endings via various inflammatory reactions (involving, for example, prostaglandins, bradykinins, endothelin, and nerve growth factor) [29]. In bone and in periosteum there are mechanosensitive fibers of the nociceptive type, which respond quickly to mechanical distortions of the tissue [29]. In bone tissue, there is a direct relationship between the autonomic and the central nervous system.

Bone marrow

Red bone marrow or myeloid tissue (yellow bone marrow, consisting mainly of adipose tissue, which determines its color) is a key component of the lymphoid system, producing the lymphocytes that form part of the body’s immune system. Myeloid cells are recruited in the presence of inflammation by the sympathetic nervous system [30]. The leukocytes and neutrophils produced by the bone marrow are released into the systemic circulation, starting from the venous sinuses of irregular caliber known as sinusoids (or vascular sinuses). Immune cells will then be recruited from the inflamed or injured site [31]. Bone marrow participates in the repair and defense of the body system for bone tissue and for all other organs and tissues.

Improving the current definition of the fascial system

Bone tissue corresponds perfectly to the definition of fascia [5]. It is able to remodel in response to mechanical stimuli, and it is in synergy with other structures of the human body, influencing the systemic health of the individual. Each osteocyte communicates with all the other osteocytes in the bone where it resides. Bone is part of the fascial continuum. As an example, consider the mechanical stimulus of a voluntary movement such as walking, where the tension felt by the epidermis of the foot passes through all the tissues to the bone, which participates in the adaptation of the whole body in a biunivocal mode through autocrine and paracrine actions. Comparing our previous definition of fascia with our current definition, we added the term “feeding,” as arterial blood nourishes the fascia and is an integral part of the fascial continuum. Regarding the concept of nurturing, the action of venous blood and lymph is inherent, these being integral parts of the definition of fascia; an adequate metabolic environment is created to best utilize the nutrients that the arteries carry [4, 6]. But it is not only the arteries that contribute to a satisfactory metabolic environment. The entire fascial system transmits substances between different tissues and between cells to inform what happens from a mechano-metabolic perspective and to facilitate an adequate mechano-transduction process [32-33]. The ability to receive information is vital to be able to adapt and survive, from the whole tissue to the single cell. We can define it as an informational “nutrition.” Finally, we added two other words to improve the definition of the band: liquids and solids. The fasciae inside the human body exist as both a solid structure and a liquid structure [4, 6]. We reiterate our previous definition of fascia, with some words added (highlighted in italics): “The fascia is any tissue that contains features capable of responding to mechanical stimuli. The fascial continuum is the result of the evolution of the perfect synergy among different tissues, liquids and solids, capable of supporting, dividing, penetrating, feeding and connecting all the districts of the body, from the epidermis to the bone, involving all the functions and organic structures. The continuum constantly transmits and receives mechano-metabolic information that can influence the shape and function of the entire body. These afferent/efferent impulses come from the fascia and the tissues that are not considered as part of the fascia in a biunivocal mode.”

## Conclusions

This article reviewed the main functions of bone and its related anatomy, as well as the capacity of bone to adapt in response to mechano-metabolic stimuli. We have emphasized skeletal relationships in relation to the systemic health of the individual, with biunivocal modalities, inserting the skeletal network into the definition of fascia. We have added in the description the term “feeding,” because the liquid bands, like the blood and the lymph, have peculiarities that allow the nourishment of different tissues. The same tissues feed on mechano-metabolic information, which is mutually exchanged with the ultimate aim of adapting and surviving. Other words added to enrich the definition of fascia are “liquids and solids,” because the fascial tissue is composed of both solid and liquid material. We believe that further research is needed to achieve a truly complete definition of fascia, in the light of pressing and constant new scientific information.
